# Government capacities and stakeholders: what facilitates ehealth legislation?

**DOI:** 10.1186/1744-8603-10-4

**Published:** 2014-01-13

**Authors:** Achim Lang

**Affiliations:** 1 Department of Politics and Public Administration, University of Konstanz, Postbox 5560, D81, Konstanz 78457, Germany

**Keywords:** EHealth, Regulation, World Health Organization, European Union, OECD, Public-private partnerships, Donors, Capacity development

## Abstract

**Background:**

Newly established high-technology areas such as eHealth require regulations regarding the interoperability of health information infrastructures and data protection. It is argued that government capacities as well as the extent to which public and private organizations participate in policy-making determine the level of eHealth legislation. Both explanatory factors are influenced by international organizations that provide knowledge transfer and encourage private actor participation.

**Methods:**

Data analysis is based on the *Global Observatory for eHealth - ATLAS eHealth country profiles* which summarizes eHealth policies in 114 countries. Data analysis was carried out using two-component hurdle models with a truncated Poisson model for positive counts and a hurdle component model with a binomial distribution for zero or greater counts.

**Results:**

The analysis reveals that the participation of private organizations such as donors has negative effects on the level of eHealth legislation. The impact of public-private partnerships (PPPs) depends on the degree of government capacities already available and on democratic regimes. Democracies are more responsive to these new regulatory demands than autocracies. Democracies find it easier to transfer knowledge out of PPPs than autocracies. Government capacities increase the knowledge transfer effect of PPPs, thus leading to more eHealth legislation.

**Conclusions:**

All international regimes – the WHO, the EU, and the OECD – promote PPPs in order to ensure the construction of a national eHealth infrastructure. This paper shows that the development of government capacities in the eHealth domain has to be given a higher priority than the establishment of PPPs, since the existence of some (initial) capacities is the *sine qua non* of further capacity building.

## Introduction

This paper analyzes the factors that enable or impede the legislation of national eHealth infrastructure. In particular, it examines the role and effects of stakeholders such as companies or private donors on the legislation of national health infrastructures. The question of whether the participation of private organizations enhances the capacity of national governments to regulate these eHealth infrastructures is raised. Furthermore, this paper asks if the participation of private organizations makes a difference for autocratic or democratic regimes, and whether the participation of private organizations increases the governing capacities of resource-poor governments, leading to more legislation.

The participation of stakeholders in policy-making is seen as an enabling instrument to successful policy implementation. It is particularly important when governments do not have the capacity to act or regulate the matter due to absent knowledge or resources. This becomes even more serious in the case of developing countries. International organizations such as the WHO, the OECD, and the European Commission strongly encourage the involvement of non-state actors in order to increase the knowledge base in policy-making. Stakeholder participation is said to compensate for the lack of government capacity by providing expertise on particular issues. However, policy scientists point out that not only does low government capacity constrain the ability to set up legislation, but also sub-system complexity. Increasing numbers of participating stakeholders in domestic policy-making and policy implementation may lead to mounting complexity concerning the policy process [1-5]. In such complex settings, regulatory decisions are less likely [6-8].

This paper seeks to shed light on this paradoxical situation and tries to determine if the participation of stakeholders increases the capacities of governments or if they increase policy-subsystem complexity. I argue that the participation of stakeholders increases government capacities to regulate health infrastructures under the condition that these governments already possess some capacities. This argument is based on the assumption that governments have to acquire expertise from other organizations or from experiences gained by providing health infrastructures or in the legislation of other branches of the economy. In order to regulate health infrastructures, governments that already have some experience in regulatory matters find it easier to set-up legislations than governments with little or no expertise.

The impact of stakeholder participation is tested in the case of eHealth^a^, which has become an important topic for the provision and coordination of health care in developed as well as developing countries. eHealth refers to “health services and information delivered or enhanced through the Internet and related technologies. In a broader sense, the term characterizes not only a technical development, but also a state-of-mind, a way of thinking, an attitude, and a commitment for networked, global thinking, to improve health care locally, regionally, and worldwide by using information and communication technology” [9]. eHealth applications include, to name just a few, electronic health records in which all diagnoses and medical treatments are stored, mobile interfaces between patients and physicians, and decision support systems that provide immediate information and feedback to physicians during consultations and treatments.

eHealth has generated high hopes within international organizations, in particular the WHO, the OECD and the European Commission. The use of ICT is expected to result in higher efficiency, increased profits, lower costs, and better quality for the user. eHealth is widely viewed as an all-purpose measure to alleviate health inequalities in developing countries and to bring health care to even the most remote villages. In industrialized OECD and EU countries, eHealth is expected to increase coordination between the specialized medical professions.

The application of ICT in health care delivery rests on the ability of governments and international organizations to provide and regulate interfaces as well as interoperability standards between hospitals, physicians, sickness funds, government agencies, and above all, patients [10]. Additionally, legislations must ensure that the use of eHealth services meets privacy requirements.

The paper proceeds as follows. The next section outlines policy instrument choice in a multi-level polity and focuses on the role of stakeholders – private enterprises, donors and public-private partnerships (PPP) – in affecting government capacity. The third and fourth sections provide information about data collection and statistical models. It is followed by an analysis of eHealth policies in 114 countries. And finally, the conclusion summarizes the findings.

### The choice of legislation in a multi-level policy regime

The choice and scope of legislations is a highly constrained enterprise that involves choices at different levels of the policy-making architecture [11]. Howlett basically distinguishes between three regime levels in a nested political setting. These levels include international regimes, the national policy regime, and the sectoral policy regime.

At the international level, generic governance modes determine macro policy goals and general implementation styles, with the effect that different governance modes generate different preferences for policy instruments. These governance modes are compatible with certain macro-policy objectives or “policy paradigms” [12]. Macro-policy paradigms are not only time-dependent, but they also depend on incorporation into international regimes. Their scope and degree of authority limits the choices available to state governments, as well as changes the preferences of domestic actors regarding different policy tools. They provide interpretive frameworks, which structure cognitive processes as well as the terminology of political communication [13]. An international regime essentially denotes a “set of implicit or explicit principles, norms, rules, and decision making procedures around which actors’ expectations converge in a given area of international relations” (Krasner 1983: 2). The basic elements of international regimes consist of institutions that regularize actor participation and expectations. Rules and norms have become central tenets of international regime literature [14-16]. Norms and rules put forth by international regimes are related to this selection process in how they affect capacity building in member states and stakeholder participation. Most, if not all international health regimes have been designed to assist member states in tackling health issues and to provide them with the means to do so. This includes advice and recommendations as well as financial assistance. Rather frequently, it also includes the participation of a diverse set of organizations in the formulation and execution of public policies.

Many international eHealth regimes have been set-up in the last two decades. The World Health Organization (WHO), the European Union (EU) and the Organization for Economic Cooperation and Development (OECD) provide the most advanced efforts to structure member state activities in the eHealth domain (Table 1).

**Table 1:** International eHealth regimes

		**WHO**	**OECD**	**EU**
**Norms**	**Origin**	Millennium development goals	Science and technology policy	Lisbon strategy for growth and jobs
	**Problem perception**	Fighting poverty and poor health; insufficient resources in developing countries	Insufficient coordination between state agencies; insufficient ICT infrastructure	Ageing societies; increasing costs
	**Objectives**	Advice and recommendations regarding knowledge/technology; cooperation between public and private actors	Technology diffusion; higher quality health care; establishing the market for eHealth solutions	Driver for economic growth; more efficient and higher quality of health care delivery; market creation
**Rules**	**Procedures**	Supporting activities of the WHO; WHO works with other IOs	Commissioned evaluation reports; workshops with member country reps.	Participation open to member states (and sometimes to stakeholders)
	**Actors**	States, stakeholders	States, stakeholders	States, stakeholders
	**Policy instruments**	Benchmarking by WHO, standard setting, coordination of standardization organizations, policy and technology advice, and monitoring	Best practices, policy and technology diffusion, and learning between member states	Establishment of standardization projects for member states, policy and technology recommendations, priority setting, and project funding

The WHO has long been assisting countries in providing and purchasing essential health technologies (WHO 2004). A particular emphasis on technology diffusion became more prominent after the elaboration of the Millennium Development Goals, which mandated that the WHO further engage in the diffusion of ICT-related health activities in developing countries [17]. In 2005, the World Health Assembly adopted the WHO eHealth resolution (WHA58.28), which noted the high salience of the eHealth issue and prospective benefits of eHealth to health care delivery, public health, health research, and health-related activities (WHA58.28: 1). Furthermore, it urged member states to develop a strategic plan, a legal framework and technical infrastructure (WHA targets) to implement eHealth services, mobilize participation and coordinate activities with stakeholders, establish national centers for coordinating activities and for benchmarking and identifying best practices (WHA58.28). IT solutions are part of a larger knowledge management approach that encompasses efforts directed at “resource-poor settings”, data standardization, aggregation and international cooperation in knowledge-sharing. In order to meet the WHA targets, the WHO intensified relationships with ICT professional associations [18,19]^b^.

In the European Union, eHealth and telemedicine entered the European agenda at the end of the 1990s in the wake of the “Lisbon strategy” for growth and jobs [20,21]. Information technology was designated to play a vital role in accomplishing the Lisbon strategy targets^c^ including the exploitation of new technologies in health care delivery (later renamed “eHealth”). In 2008, the Commission announced plans to make eHealth one of the six EU Lead Market Initiatives [22] “due to its market potential in terms of growing demand and market growth opportunities, changing demographics, disease patterns and healthcare capabilities” (p. 12). The European Commission policy strategy is embedded in significant research activities that explore issues of standardization and interoperability. The lead market initiative features activities on semantic interoperability, the development of a roadmap for necessary policy steps and the establishment of a thematic network consisting of national health ministries, business associations and professional groups.

The OECD identified technology diffusion as a major driver of economic growth, especially in developing countries^d^. Broadband policies and national broadband plans (NBP) have become key areas for the development of the internet society and e-government. The OECD has proposed recommendations regarding e-government with a strong emphasis on eHealth [23]. The OECD particularly acknowledges gains in coordination, efficiency and the quality of care by increasing the use of information and communication technologies in health care delivery [24].

Although international regimes were established under varying circumstances and promoting somewhat differing ends, their means are highly similar. All international regimes provide best practices, promote standard-setting activities and endorse the participation of private actors in the development of eHealth infrastructure and eHealth legislation. There are clear policy recommendations promoting the participation of stakeholders in the policy process. This shift in responsibility is advertised as a logical result of financial constraints on the part of international organizations and the need to incorporate (more resourceful) private actors in the policy process. It is argued that new technologies spread across developed and quickly-developing countries, leaving poor countries behind. Stakeholder participation, in this view, provides an effective means to foster technology uptake even in the poorest countries, since private organizations are able to wield the financial resources and knowledge that is often missing in developing countries [25]. As a result, all forms of stakeholder participation flourished in the last two decades and have become preeminent in the field of development policy [26].

#### 

H1: *The participation of private organizations enables governments to set-up eHealth legislations*.

The national policy regime level mediates between international regime stimuli and sectoral policy-making. Howlett argues that policy instruments are selected according to the norms and rules promoted by international regimes, but also according to the logic of the prevailing national mode of governance (Howlett 2009) [11].

At the national policy regime level, a basic distinction must be made between autocratic and democratic political regimes. It is often assumed that democratic regimes are more responsive to citizen needs and IO demands while autocratic governments act in favor of the ruling elites. There are two (not mutually exclusive) explanations as to why this is the case. Both explanations are rooted in the public choice argument that public goods such as infrastructures must be supplied by the state, since individuals are unable to provide them. In the first variant, the state’s policy regarding public infrastructure reflects the citizens’ preferences about the infrastructure and their legislation [27]. In the case of eHealth, it can be assumed that citizens prefer a regulated eHealth infrastructure since it could increase health care efficiency and bring health expertise to even the most remote areas of the country. Autocratic regimes that are not confronted with the electorate might determine not to build such an infrastructure or avoid regulating them properly [28]. Another explanation is provided by Deacon [29]. He asserts that the cost-benefit ratio for elites in an autocratic regime is high, since the elites have to bear the majority of costs attributed to public infrastructure, but reap only a small amount of the benefits. “For non-exclusive public goods, however, the elite receive only a pro-rata share of the services produced. Even if the benefits these goods confer are income elastic, the elite arguably enjoy only a tiny fraction of economy-wide benefits. The uneven capture of costs and benefits by the elite causes non-democratic governments to under-provide public goods relative to democracies” (Deacon 2003: 7). The literature on the level of legislation and regime type is still in its infancy. However, there is a strong indication that autocratic regimes have a lower level of legislation than democracies [30].

#### 

H2: *Autocratic regimes have lower levels of eHealth legislation*.

Additionally, preferences regarding market legislation may have an impact on the governance of technology uptake in national health systems. Howlett argues that government preferences in most liberal-democratic countries are currently molded according to the logic of market governance “whose goal is the efficient delivery of consumer and capital goods and services through the use of market-mechanisms” (Howlett 2009: 78) [11]. Other countries rely on other governance “modi”, in particular, corporatist or etatist forms of governance in which governments act alone or authorize societal actors to take part in the decision-making process or even delegate state authority to them [31-33]. In case of eHealth legislations, it is more likely that governments relying on market forces prefer not to regulate ICT infrastructure in health care.

#### 

H3: *Countries relying on economic liberties have lower levels of eHealth legislation*.

At the sectoral regime level, Howlett distinguishes between two interlinked sets of factors affecting policy instrument choice. First, the organizational capacity of governments to gather knowledge and other resources to execute and effect changes determines the choice and calibration of policy instruments. Second, the level of subsystem complexity involves “the number and types of actors that governments must affect in designing and implementing their programs and policies” (Howlett 2005: 43). A high degree of organizational capacity makes it likely that the government will intervene directly by providing the public good itself or by setting up legislations and monitoring agencies. Lower degrees lead to indirect interventions such as media campaigns or subsidies. The complexity of policy subsystems determines the degree of authority that can be exercised by national governments. Governments that face simple actor settings are able to directly influence actor behavior, and are therefore more likely to resort to authoritative policy instruments [7].

This functional perspective on the role of private organizations is accompanied by critical accounts that stress the dysfunctional aspects such as project proliferation and the redundancy of coordination structures. In a meta-analysis, Buse and Walt enumerate and summarize the effects of actor participation on national governments [34]. They provide evidence that the large-scale participation of donor and other private organizations distracts resources away from national health ministries and other public health authorities to record-keeping of projects and coping with the conditionality set by donor/private organizations. These distracted resources are taken away from planning efforts and may lead to “institutional destruction”, which concerns the effects large scale donor participation has on governmental capacities [35].

However, different types of stakeholder organizations have different effects on public policy. In particular, private and donor organizations seem to create negative externalities in pursuit of their financial funding and project activities [36-40]. Furthermore, donor organizations often set agenda and priorities for national health care ministries, which do not necessarily coincide with domestic demands. Shiffman reports that “funding does not correspond closely with burden” [38]. In particular, the fight against communicable diseases (e.g. Malaria, HIV/AIDS) receives an unequally higher percentage of funding than non-communicable diseases (e.g. respiratory infections), although these amount for the majority of disease prevalence and mortality [38]. He further notes that displacements from population health and health system funding towards communicable disease funding have occurred, but overall increases in funding levels have covered these developments [39]. Similarly, Esser and Bench analyzed grants to low and middle-income countries from private organizations and donors to fight the disease burden. Their study reveals that donors and private actors are not overly responsive to national patterns of disease burden, but pursue other health priorities [37]. Carmignani et al. further investigated the causes for the shift in donor priorities and concluded that “greater media coverage increases aid disbursement” (p. 18). This affects particular diseases with higher morbidity and mortality as well as infrastructure programs [36].

#### 

H4: *The participation of private organizations diverts government activities away from planning and setting up legislations*.

In contrast, PPPs receive favorable recommendations since they provide platforms for knowledge transfer that enable public authorities to learn from private organizations as well as gain experience in crafting and implementing projects. This hands-on experience provides another source of expertise that enhances public capacities [25,41].

#### 

H5: *The participation of public organizations in public-private partnerships (PPPs) enables governments to set up legislations*.

### Data

Data analysis is based on the *Global Observatory for eHealth - ATLAS eHealth country profiles* which summarize the second global survey on eHealth that was carried out from June to December 2009, and which served as the base for *The Global Observatory for eHealth Series*. The survey was developed by the *Global Observatory for eHealth* with intensive consultation and input from eHealth experts. In a way, the first survey on eHealth served as a pre-test in terms of data collection and management. A pre-test of the final questionnaire for the second global survey was carried out in March 2009 covering Canada, Lebanon, Norway, the Philippines, and Thailand.

According to the WHO Global Observatory for eHealth, 800 eHealth experts in 114 countries participated in the data collection, ranging from 5 to 15 per country [42]. The Global Observatory for eHealth implemented a variety of measures to assure the quality and validity of the expert ratings. The country experts received detailed instructions to maintain consistency in the responses and expert teams were assisted by staff from the WHO regional offices. Additionally, external information sources were used in order to validate the data and resolve contradictions. Since the country experts were required to reach consensus and come up with a single response for each question, differences in opinion had to be settled by determining which response is the most representative for the country as a whole. As a result regional variation could not be measured at the country level [43].

However, the Global Observatory for eHealth admits that “there was significant variation across Member States in the quality and level of detail in the responses, particularly for the descriptive, open-ended questions. While survey responses were checked for consistency and accuracy, it was not possible to verify the responses for every question” [43].

The questionnaire hosted a collection of three broad topics with eight items that covered the main aspects of eHealth legislation:

• *Legislation on personal and health-related data:* (1) To ensure the privacy of personally identifiable data; (2) To protect personally identifiable data specifically in electronic medical records (EMR) or electronic health records (HER).

• *Legislation for sharing health-related data between health care staff through EMR/HER:* (1) Within the same health care facility and its network of care providers, (2) With different health care entities within the country, (3) With health care entities in other countries.

• *Internet pharmacies:* (1) Legislation that allows/prohibits Internet pharmacy operations; (2) National legislation/accreditation/certification of Internet pharmacy sites; (3) Legislation that allows/prohibits Internet pharmacy purchases from other countries.

The response variable was created as an additive index (including positive entries only).

The questionnaire furthermore contained questions about eHealth expenditure per function and funding source. This is taken as an indicator of the activities of different actors such as public authorities, private organizations, donors and public-private partnerships. The questionnaire listed six main functions where different actor categories might be involved:

1. ICT equipment

2. Software

3. Pilot projects

4. Skills training

5. Ongoing support

6. Scholarships

All variables were constructed as additive indices per actor category (including positive entries only). This index covers the breadth of actor involvement in eHealth implementation and is used as a proxy for actor participation. To be sure, it would be preferable to measure the number of organizations involved in policy-making and implementation directly as well as the resources spent. However, such an indicator does not exist and is almost impossible to construct. This index provides a reasonable proxy for actor participation.

Concept specification for government capacity includes different dimensions. First, financial resources have to be considered since the gathering of information [44] and the elaboration of legislations are resource-consuming efforts. Government expenditures on health care and average annual government deficits provide indicators for financial capacities.

Experience in market legislation is another dimension that is associated with national policy regimes. It indicates whether governments rely on market mechanisms or on market restrictions. A combined legislation index from the Economic Freedom of the World database is used to indicate the intensity of government legislation. The combined index includes a regulation index for labor and capital market regulations. It tells us if a country guarantees property rights but does not restrict economic freedoms. The autocracy-democracy distinction is measured by the Polity IV score, a measure often used in comparative political studies.

In order to measure the possible autonomy of national governments, their independence from private donors or the potential overload of external donor participation, an indicator for Official Development Assistance (ODA) received (% of GNI) is included.

Regarding the regulatory and public policy challenge in eHealth, two indicators will be included in the analysis. First, physician density includes the idea that eHealth in health care is set-up to achieve better coordination between health care providers and to relieve cost pressures from health care systems. Population density, in contrast, refers to the claim that health care should be delivered even in the most remote areas of a country.

Regarding missing values, multiple imputations from the “Amelia” package [45,46] in the programming environment R [47] created 25 different data sets in order to alleviate the effects of the imputed missing values on the regression model. The final dataset contains the average values of all imputed datasets^e^.

## Methods

Data analysis was carried out using hurdle Poisson models for count data. These are two-component models that consist of a truncated Poisson model for positive counts and a hurdle component model with a binomial distribution for zero or greater counts [48,49]. The hurdle component analyzes the binary part of the model for which the dependent variable is either zero or has a positive value. It therefore captures the question of if a country has health legislation at all, irrespective of the total amount of legislation. The Poisson model considers the count part of the model and analyzes how many legislations countries have [49]. The analysis is based on the package “pscl” [50] contained in the R programming environment.

The principal idea for the hurdle Poisson model is that there are two different processes at work. The first process causes the absence or presence of eHealth legislations, while the second process influences the number of eHealth legislations. The probability function for a hurdle Poisson model is built up accordingly. The binomial distribution is used to model the absence and presence of eHealth legislations, and a Poisson distribution is used for the counts [51]:

$$f\left(y,\beta ,\gamma \right)=\left\{\begin{array}{c} f_{\mathrm{binomial}}\left(y=0;\gamma \right)\\ \left(1-f_{\mathrm{binomial}}\left(y=0;\gamma \right)\right)\times \frac{f_{\mathrm{Poisson}}\left(y,\beta \right)}{1-f_{\mathrm{Poisson}}\left(y=0;\beta \right)} \end{array}\right)\;\begin{array}{c} y\;=0\\ y\;>0 \end{array}$$

It follows that the probability of measuring no legislation is modeled with a binomial distribution, where *π*_*i*_ is the probability that *y*_*i*_ = 0. *π*_*i*_ is modeled in terms of covariates *Z* and regression parameters *γ*[51]:

$$\pi_{i}=\frac{e^{v+\gamma_{1}\times Z_{i1}+\ldots \gamma_{q}\times Z_{\mathrm{iq}}}}{1+e^{v+\gamma_{1}\times Z_{i1}+\ldots \gamma_{q}\times Z_{\mathrm{iq}}}}$$

In order to measure a non-zero count, the government needs to cross a hurdle to produce a non-zero value (create legislation) *and* the Poisson count process has to exclude the probability of zero values, which is called a zero-truncated Poisson distribution. The second part in the first equation means that the probability of measuring a value greater zero equals the probability that it is not a zero multiplied with the probability determined by a zero-truncated Poisson. The mean of the Poisson distribution is modeled in the following way [51]:

$$\mu_{i}=e^{a+\beta_{1}\times X_{i1}+\ldots \beta_{q}\times Z_{\mathrm{iq}}}$$

## Results

The number of eHealth legislations is unevenly distributed between OECD/EU and developing countries (see Table 2). OECD/EU countries have almost four different eHealth legislations on average (3.79). In contrast, countries in other parts of the world have only slightly more than one legislation in the eHealth domain (1.10).

**Table 2:** Descriptive statistics

**Indicator**	**Mean**	**Std.**	**Min.**	**Max.**	**Source**
eHealth legislation (sum of eHealth legislation)	1.81	1.81	0.0	7.0	Global observatory for eHealth - ATLAS eHealth country profiles
eHealth legislation (OECD/EU countries, N = 29)	3.79	1.66	1	7	
eHealth legislation (developing countries, N = 86)	1.10	1.26	0	5	
Public actors (no. of eHealth functions)	3.64	2.18	0.0	6.0	Global observatory for eHealth - ATLAS eHealth country profiles
Private actors (no. of eHealth functions)	1.63	2.15	0.0	6.0	Global observatory for eHealth - ATLAS eHealth country profiles
Donors (no. of eHealth functions)	2.60	2.40	0.0	6.0	Global observatory for eHealth - ATLAS eHealth country profiles
Public-private partnerships (no. of eHealth functions)	1.25	1.80	0.0	6.0	Global observatory for eHealth - ATLAS eHealth country profiles
Government expenditures on health care (% total expenditures on health)	56.63	18.38	10.50	87.70	WHO World health statistics
Government surplus/deficit (average from 2000–2010)	−0.91	4.10	−11.56	14.36	World development indicators
Labor and credit market regulations (high values = less regulation and restriction)	7.23	1.11	4.50	9.57	Economic freedom of the world http://www.freetheworld.com/
Net official development assistance (ODA) received (% of GNI)	5.367	10.16	−18.33	73.48	World development indicators
Autocracy-democracy (Polity IV score)	4.12	6.30	−10	10	Polity IV Project http://www.systemicpeace.org/polity/polity4.htm
Physicians (per 10,000 inhabitants)	15.78	14.207	0.50	53.50	WHO World health statistics
Population density (km^2^)	197.88	694.89	2.00	7202.00	World telecommunication/ICT indicators database

Note: sum of eHealth legislation means the number of different pieces of legislation that have been enacted (*Legislation on personal and health-related data:* (1) To ensure the privacy of personally identifiable data; (2) To protect personally identifiable data specifically in EMR or HER. *Legislation for sharing health-related data between health care staff through EMR/HER:* (1) Within the same health care facility and its network of care providers, (2) With different health care entities within the country, (3) With health care entities in other countries. *Legislation on Internet pharmacies:* (1) Legislation that allows/prohibits Internet pharmacy operations, (2) National legislation/accreditation/certification of Internet pharmacy sites, (3) Legislation that allows/prohibits Internet pharmacy purchases from other countries). The mean value of 1.81 indicates that on average about 2 pieces of legislation have been enacted. The no. of eHealth functions is an additive index that covers the whole range of functions in which a particular actor, such as donors or PPPs, participates (ICT equipment, software, pilot projects, skills training, ongoing support, scholarships, etc.). The mean value of 3.64 for public actors indicates that on average public actors are actively implementing about four of these functions in each country.

This paper seeks to analyze the drivers of eHealth legislations. The main argument concerns the effects of private actor participation in government legislation of eHealth and whether political regimes and governmental capacities provide different leverage for private actors.

**Figure 1:**
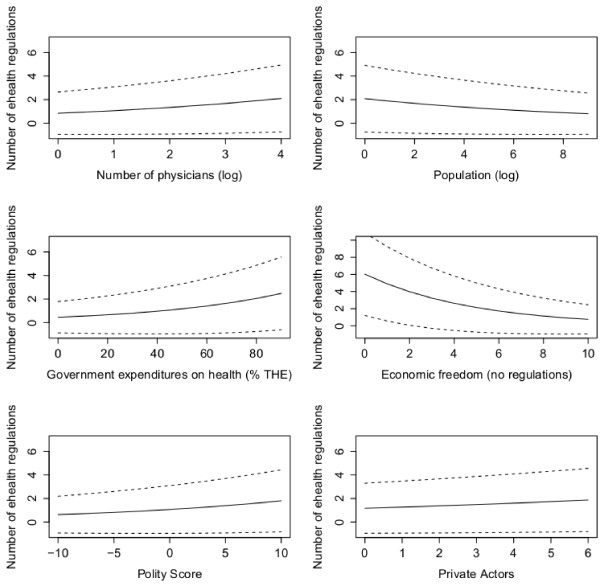
**Effects plots for Model 4.** Note: Predicted effects over a range of values with 95% pointwise confidence bands for the mean response shown as dotted lines.

At first, however, factors closely linked with socio-economic challenges regarding the use of eHealth in health care delivery are considered (see Model 1 in Table 3). Such factors include the number of physicians in a country and population density. Regarding the number of physicians, the coefficients indicate significant and positive effects on eHealth policies in the model containing all countries, while coefficients for the non-OECD/EU countries are positive, yet not significant (see Figure 1). The coefficients for the zero component have positive values and are highly significant in every model. This suggests that legislation is triggered by the need to coordinate the medical profession.

**Table 3:** Hurdle Poisson models for all countries and for non-OECD/EU countries (response variable: eHealth legislation, standard errors in parentheses)

	**All countries**				**Non-OECD/EU countries**			
	**Model 1**	**Model 2**	**Model 3**	**Model 4**	**Model 5**	**Model 6**	**Model 7**	**Model 8**
	Count model coefficients (truncated poisson with log link)							
(Intercept)	0.03 (0.40)	0.15 (0.69)	0.82 (0.12)***	0.50 (0.78)	0.33 (0.49)	1.36 (1.13)	0.18 (0.24)	1.81 (1.15)
Physicians per 10,000 inhabitants (log)	0.45 (0.10)***			0.23 (0.11)*	0.16 (0.12)			0.13 (0.13)
Population Density km^2^ 2007 (log)	−0.12 (0.06)*			−0.11 (0.06)^#^			−0.11 (0.10)		
Government expenditures on health		0.02 (0.01)***		0.02 (0.01)*		0.03 (0.01)**		0.02 (0.01)*	
Government surplus/deficit (average from 2000–2010)		0.01 (0.02)				−0.04 (0.05)			
Economic freedom in labor and credit market (less regulation)			−0.23 (0.09)**	−0.21 (0.09)*		−0.51 (0.18)**		−0.52 (0.19)**	
ODA received		−0.01 (0.02)				−0.02 (0.03)			
Autocracy-democracy (Polity IV score)		0.07 (0.02**)		0.05 (0.02)*		0.04 (0.03)		0.04 (0.03)	
Public sector activity		0.08 (0.06)				0.09 (0.11)			
Donor activity			−0.17 (0.04)***	−0.05 (0.05)			−0.14 (0.07)^#^	−0.08 (0.09)	
Public-private partnership activity			0.05 (0.05)				0.35 (0.09)***	0.32 (0.10)***	
Private sector activity			0.11 (0.04)*	0.08 (0.04)*			−0.18 (0.09)*	−0.12 (0.10)	
	Zero hurdle model coefficients (binomial with logit link)								
(Intercept)	−0.12 (0.78)	1.51 (1.57)	1.14 (0.35)***	2.31 (1.69)	−0.16 (0.77)	1.33 (1.56)	0.43 (0.39)	1.71 (1.61)	
Physicians per 10,000 inhabitants (log)	0.84 (0.17)***			0.97 (0.22)***	0.66 (0.18)***			0.79 (0.22)***	
Population density km^2^ 2007 (log)	−0.07 (0.18)			−0.08 (0.21)	−0.05 (0.18)				
Government expenditures on health		0.02 (0.02)		0.02 (0.02)		0.01 (0.02)		0.02 (0.02)	
Government surplus/deficit (average 2000–2010)		−0.03 (0.06)				−0.03 (0.06)			
Economic freedom in labor and credit market (less regulation)		−0.37 (0.26)		−0.54 (0.30)^#^		−0.32 (0.26)		−0.50 (0.30)^#^	
ODA received		−0.05 (0.03)^#^				−0.03 (0.03)			
Autocracy-democracy (Polity IV score)		0.12 (0.04)**		0.12 (0.05)**		0.07 (0.04)^#^		0.08 (0.05)^#^	
Public sector activity		0.23 (0.12)^#^				0.23 (0.12)^#^			
Donor activity			−0.24 (0.10)*	0.03 (0.13)			−0.07 (0.11)	0.03 (0.13)	
Public-private partnership activity			0.32 (0.16)^#^				0.35 (0.17)*	0.26 (0.18)	
Private sector activity			0.08 (0.12)	0.04 (0.14)			−0.04 (0.14)	−0.07 (0.16)	
N	114	114	114	114	84	84	84	84	
Log-likelihood	−180.9	−176.2	−196.8	−162.7	−114.6	−108.2	−113.7	−97.19	
Df	6	14	8	16	6	14	8	16	

***p < 0.001 **p < 0.01 *p < 0.05 ^#^p < 0.1.

Population density clearly shows a negative relationship with legislation. Higher densities are accompanied by lower levels of eHealth legislation. However, this effect is only present in the all-countries model, but vanishes in the model containing only non-OECD/EU countries. Thus, the main rationale for applying eHealth is not so much bringing health care delivery to every part of the country, but in making health care more efficient and in easing coordination between health care providers.

**Figure 2:**
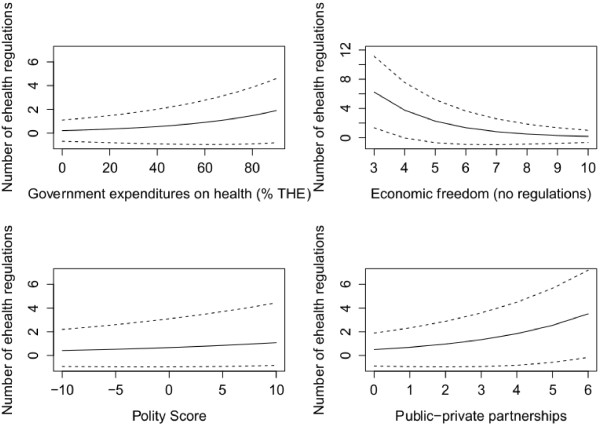
**Effects plots for Model 8.** Note: Predicted effects over a range of values with 95% pointwise confidence bands for the mean response shown as dotted lines.

Model 2 considers indicators of government capacity (see Table 3). Government expenditures on health care have a positive and significant effect in all models and imply that financial assets are the baseline for all government activities. In contrast, recent government deficits as well as a dependency on foreign aid had no impact on eHealth legislations. The level of economic freedom granted to other sectors of the economy measures the national policy style with concern to the legislation of economic activity. Economic freedoms and legislation seem to be at odds since there is a negative impact on the number of eHealth legislations. This effect is consistent in all models and holds true for developed as well as developing countries (see Figures 1 and 2). An example is the high level of economic freedom in Switzerland compared with lower levels in Germany (see Table 4). Compared to other European countries, Switzerland developed an eHealth strategy rather late in 2007. Since Switzerland is a federal state and health care delivery is dominated by private or local public health care providers, the Swiss central government lacked expertise in coordinating and regulating large infrastructure projects, in particular within the health care sector [52]. The German central government, in contrast, modernized legislation in health care delivery that introduced telematic devices in 2003. The new legislation set up the institutional framework for the subsequent implementation of the electronic health card [53]. The legal guidelines regarding eHealth are modeled after previous large-scale innovation policy projects in which the federal government was the driving force behind technology innovation. The new legislation lists the entire structure and members of the implementation organization as well as the funding scheme and privacy requirements [54]. Additionally, the organization’s structure allowed for the participation of a large number of NGOs such as health insurance associations, chambers of the medical profession or ICT business associations.

**Table 4:** Statistics for selected countries

	**No. eHealth policies**		**Government expenditures on**		**Polity score**		**Economic freedom**	
			**health (% THE)**					
**Country**	**Value**	**Rank**	**Value**	**Rank**	**Value**	**Rank**	**Value**	**Rank**
CHE	2	32	59.6	55	10	1	8.8	10
GER	5	5	75.7	20	10	1	6.7	78
TKM	1	49	52.4	68	−9	111	6.2	92
TUR	3	23	75.2	24	7	53	5.8	99

Another comparison illustrates the differences between democracies and autocracies in legislating eHealth. Autocracies are less responsive to demands by the international community and their electorate to set-up adequate eHealth legislations that enable health care providers to exchange patient information and guarantee data protection and privacy. An example is Turkmenistan. In Turkmenistan, the autocratic government has not identified eHealth as a major policy area. Neither a policy framework nor a strategic plan is in place that acknowledges some need to coordinate the medical profession or health care delivery in this vast country. This lack of policy is in line with the low efforts being made to modernize the government and provide an ICT infrastructure for public authorities [55]. Only recently has there been some movement as international collaborations regarding e-government, e-education and e-health have been initiated. India is the primary collaborator in these matters (The Economic Times, Sept-18- 2012). In contrast, Turkey has established an eHealth project and strategy in support of the larger “Health Transformation Project” of 2003. The eHealth strategy was designed as a top-down process to establish guidelines and adopt interoperability standards. The responsible Turkish Ministry of Health envisaged increasing efficiency and coordination of the delivery of health care, thereby reducing costs to the health care system. However, the eHealth project was slowed down due to a lack of expertise in standards and operability issues. As a result, the Turkish Ministry of Health made a request to the ITU to provide assistance in the implementation of the eHealth project [56]. Since January 2009, the national health information system connects 100% of all Turkish hospitals that utilize a common electronic patient record system [57].

Models 3 and 7 include indicators of the policy subsystem. They explore the effect of actor participation on the degree of eHealth legislation. As it turns out, donors, private actors and public-private partnerships make a difference in the policy-making process. However, their involvement differs for OECD/EU and most developing countries. In non-OECD/EU countries, private organizations, donors and PPPs have significant effects on eHealth legislations, but in opposite directions. The participation of donors decreases eHealth policy-making (see Figure 2). However, the effects fall short of reaching an adequate significance level if one controls for capacity indicators. This allows an interpretation that donor participation occurs essentially in countries with low capacities. Their participation is not directed toward the establishment of a legal framework that guarantees interoperability but on the development of their projects. The same holds true for private actor involvement, although this effect is also (slightly) not significant when controlled for the capacity variables. An interpretation might be that private organizations and donors are principally interested in carrying out their projects undisturbed by domestic government interference. However, government legislation increases with higher capacities, thus softening business pressures to neglect eHealth legislations. In contrast, PPPs have a positive effect on the number of eHealth policies, but only in non-OECD/EU countries, while there is no effect in developed countries (see Figure 2). Thus, PPPs enable knowledge transfers from private organizations to public authorities in developing countries.

In order to analyze the effects of PPPs and donor involvement more deeply, Table 5 provides predicted means from the count component of model 8. It shows the predicted effects of PPPs for different political regimes and levels of government capacity. Regarding the difference between autocracies and democracies, PPP effects for autocracies is about half of the effects for democracies (e.g. 1.18 legislations vs. 2.24 for three PPPs). This allows for the interpretation that democracies find it easier to transfer knowledge out of PPPs than autocracies. Similarly, government capacities in health care as measured by government expenditures and the amount of legislations in other sectors of the economy give us a striking picture. Government capacities increase the effect of PPPs by about 40% (for the 9^th^ decile of countries with very high capacities). If governments have extensive experience in regulating other sectors, then the additional effect of having PPPs increase the number of eHealth legislations also by about 40%. This means that there is an exceptionally high impact of PPPs when governments already have some expertise in the health domain or in regulating other sectors.

**Table 5:** Interaction of PPP involvement with democracy, economic regulation and government health expenditures on eHealth legislation in developing countries

	**Polity score –**		**Government expenditures on**		**Labor and credit market**	
	**autocracy vs. democracy**		**health care (% of THE)**		**regulations**	
**PPP**	**Low polity score -**	**High polity score -**	**Low govern.**	**High govern.**	**Many labor and credit**	**Few labor and credit**
	**Autocracy**	**Democracy**	**expenditures on health**	**expenditures on health**	**market regulations**	**market regulations**
**(sum)**	**(1**^**st **^**decile)**	**(9**^**th **^**decile)**	**(1**^**st **^**decile)**	**(9**^**th **^**decile)**	**(1**^**st **^**decile)**	**(9**^**th **^**decile)**
0	0.60	0.85	0.50	1.18	1.41	0.28
1	0.75	1.17	0.44	1.63	1.95	0.39
2	0.94	1.62	0.39	2.26	2.70	0.54
3	1.18	2.24	0.34	3.13	3.74	0.74
4	1.50	3.10	0.30	4.32	5.17	1.03
5	1.96	4.28	0.26	5.98	7.15	1.42
6	2.61	5.92	0.23	8.27	9.89	1.96

Macro-political regime characteristics towards more democracy increase the effects of PPPs. However, regime characteristics are conceptually and empirically more distant than actual (sectoral) government capacities or cross-sectoral experience in regulating economic matters. These closer factors provide a more fruitful setting in which knowledge transfer and knowledge creation works better than in capacity-poor settings.

To sum up, the main arguments of this essay referred to the participation of private organizations in regulating national health infrastructures. It was hypothesized that their participation in domestic policy-making enhances the capacity of national governments to regulate these infrastructures. This view is consistent with the expectations and declarations of IOs such as the WHO. In contrast, scholars of public policy stress that private organizations prefer to lobby for less legislation. This should be particularly prevalent in countries where governments have low governing capacities.

As it turns out, the capacity of governments has a positive effect on the amount of eHealth legislation. The more resources governments command, the more experience governments have with legislation of other sectors of the economy, the more likely they are able to set-up eHealth legislation.

Sub-system complexity adds to the explanation of variation between developing countries. Private/business actor participation has a negative effect on eHealth legislations while PPPs increase the regulatory level. An explanation for this result is that business interests in general do not prefer governmental legislation, but (actively) try to avoid it. In contrast, PPPs especially add to the expertise of non-OECD/EU governments. This is an indication for knowledge transfer, as propagated by the WHO. Experience with legislation in other sectors of the economy raises legislation levels. This is in line with reasoning about knowledge transfer within government.

Theoretically, my arguments add some new insights to the literature of policy instrument choice. This paper considers the two main determinants of policy instrument choice, namely governmental capacities and sub-system complexity – and evaluates the degree of interrelatedness in the context of different settings. Governing capacities are a necessary and sufficient condition for the set-up of legislations and other policy instruments. This holds true for all contexts. Sub-system complexity, however, is essentially a sufficient condition since it constrains and enables policy instrument choice. Sub-system complexity seems to have various dimensions, of which one is the actor category. Distinctions must be made between actor categories that have a constraining effect, such as private enterprises and donor organizations, and actors such as PPPs that enable legislation. PPPs have the strongest influence in capacity-rich settings, in which governments have experience in regulatory activities as well as in health care. Furthermore, democratic regimes are more responsive to PPPs than autocratic regimes. PPPs enhance government capacities disproportionally in democratic regimes compared to autocratic regimes. In addition, autocracies provide fewer access points for private interests which increases the likelihood that government activities are diverted to other areas and legislation is avoided. This finding holds true for private enterprises as well as donor organizations.

At this point, it is necessary to point out the limits of this study and address further research directions that might improve the models. Theoretically, the study left out diffusion effects between countries. The WHO, the OECD and the EU provide platforms and communication channels that enable governments to learn from other countries’ experiences. However, there might be more direct links between countries that are not mediated by IOs.

Another point that has not been raised is the question of if some types of health care systems are more probable to adopt eHealth legislations than other types. Three reasons account for not integrating health system variables into the hurdle models. First, there are many typologies and no consensus as to which typology performs best. Second, these typologies typically account for OECD countries and partition them into two broad categories: Bismarck and Beveridge health care systems. However, there is no classification that involves most of the non-OECD world. Third, there is no theoretical model that allows for specifying the differential effects of health care systems on eHealth. It remains unclear why Bismarck and Beveridge health care systems should differ in their adoption rates. However, this is an important topic that directly relates to the question of technology uptake in health care systems and should be analyzed in future studies.

## Conclusions

This paper has examined a series of research questions that address the legislation of national eHealth infrastructure. Does the participation of private organizations in developing national health infrastructures enhance the capacity of national governments to regulate these infrastructures? Does the participation of private organizations make a difference for autocratic or democratic regimes? What effect does the participation of private organizations have on governments with low or high governing capacities?

As it turns out, governing capacity is the key to understanding the level of eHealth legislation in developed as well as developing countries. More capacities and experience on the part of governments leads to more eHealth legislation. Furthermore, better equipped governments are less affected by private organizations that are, in general, less enthusiastic about legislations constraining their activities. In contrast, more resourceful governments are better able to make the most of PPPs.

These results yield some practical implications for the organization of eHealth and development policy. All international regimes – the WHO, the EU, and the OECD – promote the participation of private organizations in order to ensure the construction of national eHealth infrastructure. This paper shows that the development of (government) capacities in the eHealth domain has to be given a higher priority than the participation of private organizations, since the existence of some (initial) capacities is the *sine qua non* of further capacity building. The best way to prevent the capture of government by private actors is to develop expertise within public organizations. External monitoring by international organizations might be an additional mechanism of institutional governance but it provides no substitute for the capacities of national governments.

Additionally, as Buse and Waxman point out, “partnerships […] clearly require improved systems of institutional governance. Systems should be established within public sector agencies to ensure that the greatest possible importance is attached to protecting the public’s interest” (Buse & Waxman, 2001: 752). Institutional development not only leads to less government capture but also facilitates knowledge transfer in PPPs. Public organizations find it easier to learn from private organizations if they already possess some experience in that area or have sufficient resources on hand.

## Endnotes

^a^The term and concept eHealth has emerged as a topic in medicine and public health in the last decade, but has not entered the social sciences yet. A systematic review of published definitions by Oh et al. reveals that there are more than 50 explicit definitions that capture the use of technology for health care delivery “as a means to expand, to assist, or to enhance human activities“ [9]. Frequently, eHealth definitions also include notions of commerce and business activities indicating that the term emerged from non-medical professionals and now replaces the older term “telemedicine”, which was originally used by the medical profession [10]. According to Oh and colleagues, the most commonly cited definition is the one developed by Eysenbach.

^b^Among them are the Healthcare Information and Management Systems Society (HIMMS), the International Medical Informatics Association (IMIA), and the International Society for Telemedicine & eHealth (ISfTeH).

^c^The European Commission bundled all measures regarding information society into a separate action plan, the *eEurope 2002: An information society for all*. It focused on a cheaper, faster and more secure Internet, an increase of investment in people and skills, and stimulating Internet use [24]. In 2006, the i2010 initiative was launched in order to facilitate and contribute to the implementation of the eHealth Action Plan. Its goal is to provide expert advice for the overarching i2010 High-Level Group [25].

^d^In 1996, the Development Assistance Committee of the OECD demanded that development partnerships entail technology transfer programs between developing countries and their development partners [27]. Furthermore, the OECD acknowledged the UN Millennium Development Goals and searched for ways to deal with poverty reduction in developing countries. The OECD worked together with the International Monetary Fund, the World Bank and the UN to relieve poverty in the world by promoting technology uptake and diffusion [28].

^e^Missing values have been replaced by estimated values for these variables: Government surplus/deficit (20.2% missing values), Labor and credit market regulation (20.2%), Net official development assistance (ODA) received (27.2%), Autocracy-Democracy (7.9%), and Physicians (0.9%).

## Competing interests

The author declares that he has no competing interests.

## References

[B1] Buse K Walt G Global public-private partnerships: part I-a new development in health? Bull World Health Organ 2000 78 549 561 10885184 PMC2560730

[B2] Buse K Walt G Global public-private partnerships: part II-what are the health issues for global governance? Bull World Health Organ 2000 78 699 709 10859865 PMC2560757

[B3] Buse K Waxman A Public-private health partnerships: a strategy for WHO Bull World Health Organ 2001 79 748 754 11545332 PMC2566497

[B4] Richter J Public-private partnerships for health: a trend with no alternatives? Development 2004 47 43 48

[B5] Mackintosh M Chaudhuri S Mujinja P Can NGOs regulate medicines markets? social enterprise in wholesaling, and access to essential medicines Glob Health 2011 7 4 10.1186/1744-8603-7-4 PMC3058036 21356076

[B6] Howlett M Ramesh M Patterns of policy instrument choice: policy styles, policy learning and the privatization experience Review of Policy Research 1993 12 3 24

[B7] Howlett M Ramesh M Perl A Studying public policy: policy cycles and policy subsystems 2009 Oxford: Oxford University Press

[B8] Potrafke N Does government ideology influence deregulation of product markets? empirical Evidence from OECD countries Public Choice 2010 143 135 155 10.1007/s11127-009-9494-z

[B9] Eysenbach G What is e-health? J Med Internet Res 2001 3 e20 10.2196/jmir.3.2.e20 11720962 PMC1761894

[B10] WHO Global Observatory for eHealth Building foundations for ehealth: progress of member states 2006 Geneva: WHO

[B11] Howlett M Governance modes, policy regimes and operational plans: a multi-level nested model of policy instrument choice and policy design Policy Sci 2009 42 73 89 10.1007/s11077-009-9079-1

[B12] Hall PA Policy paradigms, social learning, and the state: the case of economic policymaking in Britain Comparative Politics 1993 25 275 296 10.2307/422246

[B13] Dougherty JE Pfaltzgraff RL Contending theories of international relations: a comprehensive survey 2001 New York: Longman

[B14] Levy ML Young OR Zürn M The study of international regimes 1995 Laxenburg (Austria): International Institute for Applied Systems Analysis (Working Paper)

[B15] Young OR International regimes: problems of concept formation World Politics 1980 32 331 356 10.2307/2010108

[B16] Young OR International regimes: toward a new theory of institutions World Politics 1986 39 104 122 10.2307/2010300

[B17] UN Millennium Project Investing in development: a practical plan to achieve the millennium development goals 2005 New York: Earthscan

[B18] Geissbuhler A Haux R Kwankam SY Towards health for all: who and IMIA intensify collaboration Methods Inf Med 2007 47 503 505 17938770 10.1160/me5006

[B19] Kay M Santos J Report on the world health organization global observatory for ehealth strategic planning workshop, april 2008 Methods Inf Med 2007 46 503 505 18690371

[B20] Commission E Lisbon strategy evaluation document. Commission staff working document 2010 European Commission: Brussels

[B21] Commission E Looking beyond tomorrow: scientific research in the European union 2004 European Commission: Brussels

[B22] Commission E Lead market initiative for Europe: mid-term progress report 2009 European Commission: Brussels

[B23] Organisation for Economic Co-operation and Development National braodband plans 2011 Paris: OECD

[B24] Hofmarcher MM Oxley H Rusticelli E Improved health system performance through better care coordination 2007 Paris: OECD

[B25] Reich MR Reich MR **Public-private partnerships for public health** Public-private partnerships for public health 2002 Cambridge, Massachusetts: Harvard University Press 1 18

[B26] Lucas AO Reich MR Public-private partnerships: illustrative examples Public-private partnerships for public health 2002 Cambridge, Massachusetts: Harvard University Press 19 39

[B27] Farzin YH Bond CA Democracy and environmental quality J Dev Econ 2006 81 213 235 10.1016/j.jdeveco.2005.04.003

[B28] Mukherjee S Chakraborty D Is environmental sustainability influenced by socioeconomic and sociopolitical factors? cross-country empirical evidence Sustain Dev 2010 DOI: 10.1002/sd.502

[B29] Deacon RT Dictatorship, democracy, and the provision of public goods: working paper 2003 San Diego: Department of Economics, University of California

[B30] Bertelli AM Whitford AB Perceiving credible commitments: how independent regulators shape elite perceptions of regulatory quality Br J Polit Sci 2009 39 517 537 10.1017/S0007123409000623

[B31] Grant W Streeck W, Schmitter PC Private Organizations as Agents of Public Policy: the Case of Milk Marketing in Britain Private interest governments 1985 London/Newbury Park/New Delhi: Sage 182 196

[B32] Streeck W, Grote JR, Schneider V, Visser J Governing interests: business associations facing internationalization 2006 London: Routledge

[B33] Streeck W Schmitter PC Community, market, state - and associations? The prospective contribution of interest governance to social order Eur Sociol Rev 1985 1 119 138

[B34] Buse K Walt G An unruly melange? Coordinating external resources to the health sector: a review Soc Sci Med 1997 45 449 463 10.1016/S0277-9536(96)00365-6 9232739

[B35] Morss ER Institutional destruction resulting from donor and project proliferation in Sub-Saharan African countries World Dev 1984 12 465 470 10.1016/0305-750X(84)90024-X

[B36] Carmignani F Lordan G Tang KK Does donor assistance for HIV respond to media pressure? Health economics 2012 21 18 32 22555998 10.1002/hec.2776

[B37] Esser DE Bench KK Does global health funding respond to recipients’ needs? Comparing public and private donors’ allocations in 2005–2007 World Dev 2011 39 1271 1280 10.1016/j.worlddev.2010.12.005

[B38] Shiffman J Donor funding priorities for communicable disease control in the developing world Health Policy Plan 2006 21 411 420 10.1093/heapol/czl028 16984894

[B39] Shiffman J Has donor prioritization of HIV/AIDS displaced aid for other health issues? Health Policy Plan 2008 23 95 100 18156161 10.1093/heapol/czm045

[B40] Giri A Khatiwada P Shrestha B Chettri RK Perceptions of government knowledge and control over contributions of aid organizations and INGOs to health in Nepal: a qualitative study Glob Health 2013 9 1 10.1186/1744-8603-9-1 PMC3599768 23327564

[B41] Shortell SM Zukoski AP Alexander JA Bazzoli GJ Conrad DA Hasnain-Wynia R Sofaer S Chan BY Casey E Margolin FS Evaluating partnerships for community health improvement: tracking the footprints J Health Polit Policy Law 2002 27 49 92 10.1215/03616878-27-1-49 11942419

[B42] Global Observatory fo eHealth Global Observatory for eHealth - ATLAS eHealth country profiles 2011 Geneva: WHO

[B43] WHO Global Observatory fo eHealth Management of patient information: trends and challenges in member states 2012 Geneva: WHO

[B44] Hood CC The tools of government 1983 London/Basingstoke: Macmillan

[B45] Honaker J King G Blackwell M Amelia II: a program for missing data J Stat Softw 2011 45 1 47

[B46] Honaker J King G Blackwell M The Amelia package, version 1.6.3 2012 Harvard University http://gking.harvard.edu/amelia

[B47] R Development Core Team R: A language and environment for statistical computing 2012 Vienna, Austria: R Foundation for Statistical Computing http://www.r-project.org

[B48] Loeys T Moerkerke B De Smet O Buysse A The analysis of zero-inflated count data: beyond zero-inflated poisson regression Br J Math Stat Psychol 2012 65 163 180 10.1111/j.2044-8317.2011.02031.x 21950803

[B49] Zeileis A Kleiber C Jackman S Regression models for count data in R J Stat Softw 2008 27 1 25

[B50] Jackman S The pscl package, version 1.04.4 2007 Political Science Computational Laboratory, Stanford University http://cran.r-project.org/web/packages/pscl/index.html

[B51] Zuur AF Ieno EN Walker NJ Saveliev AA Smith GM Mixed effects models and extensions in ecology with R 2009 New York: Springer

[B52] Bundesamt für Gesundheit (BAG) “Strategie eHealth” Schweiz 2007 Bern: Bundesamt für Gesundheit

[B53] Lang A Mertes A Die Einführung der elektronischen Gesundheitskarte in Deutschland: der Einfluss von Interessenpositionen und Sektorzugehörigkeit auf die Entstehung des Implementationsnetzwerks Das Gesundheitswesen 2011 73 e12 e20 10.1055/s-0029-1246177 20169528

[B54] Lang A Mertes A Bauer JM, Lang A, Schneider V Governance of Large Innovation Projects: The Implementation of the Electronic Health Card in Germany Innovation policy and governance in high-tech industries: the complexity of coordination 2012 Berlin, New York: Springer 245 260

[B55] Gričar J Innovative cross-border eRegion development: possible directions and impact Organizacija 2007 40 86 96

[B56] Mandil S Review of and recommended improvements to Turkey eHealth Strategy 2004 Geneva: ITU

[B57] Dogac A Hülür U Caylan AK Yuksel M Heywood J Country brief: Turkey 2010 Ankara/Bonn: empirica - eHealth Strategies

